# Real-world evidence data on the monoclonal antibody erenumab in migraine prevention: perspectives of treating physicians in Germany

**DOI:** 10.1186/s10194-021-01344-1

**Published:** 2021-11-06

**Authors:** Andreas Straube, Philipp Stude, Charly Gaul, Katrin Schuh, Mirja Koch

**Affiliations:** 1 Department of Neurology, University Hospital LMU, Ludwig-Maximillians-University, 81377 Munich, Germany; 2 Neurological Practice Dr. Stude, Bochum, Germany; 3 Headache Center Frankfurt, Frankfurt a. Main, Germany; 4 Clinical Research Neuroscience, Novartis Pharma GmbH, Roonstrasse 25, 90429 Nürnberg, Germany; 5 Global Medical Affairs Neuroscience, Novartis Pharma AG, Fabrikstrasse 2, 4056 Basel, Switzerland

**Keywords:** Erenumab, Prophylactic treatment, Migraine, Real-world evidence

## Abstract

**Background:**

Erenumab, the first-in-class fully human monoclonal antibody targeting the calcitonin gene-related peptide receptor, was shown to be efficacious and safe for the prophylactic treatment of migraine in adults in randomized clinical trials. Large-scale, real-world evidence in multi-centre settings is still needed to confirm these results. Erenumab patient profiles outside clinical trials and physicians’ treatment patterns, as well as data from patients treated in Germany, a severely impacted population, are not published yet.

**Methods:**

TELESCOPE was a multi-centre survey gathering real-world data from 45 German headache centres between July 2019 and December 2019. The project consisted of two parts. In the first part, treating physicians shared their experiences on current erenumab treatment with regard to patient profiles, treatment patterns and treatment responses. In the second part, a retrospective chart review was conducted of 542 migraine patients treated with erenumab for at least three months. Treatment responses focused on various aspects of patients’ quality of life.

**Results:**

The analysis of 542 patients’ charts revealed that three-month treatment with erenumab significantly reduced monthly headaches, migraine and acute medication days. Furthermore, headache intensity and frequency were reduced in over 75 % and accompanying aura in 35 % of patients. The clinical global impression scale revealed a general improvement in 91 % of patients. According to the treating physicians’ professional judgement, 83 % of patients responded to erenumab and 80 % were satisfied with the treatment. Physicians evaluated restricted quality of life, the number of monthly migraine days and previous, prophylactic treatments as the main components of the current patient profile for monoclonal antibody recipients. Based on the assessment of physicians, erenumab reduced migraine symptoms in 65 % and increased quality of life in more than 75 % of their patients.

**Conclusions:**

TELESCOPE confirms positive treatment responses with erenumab shown in clinical trials in a real-world multi-centre setting. The results show consistently positive experiences of physicians utilizing erenumab in clinical practice and underline that therapy with this monoclonal antibody is effective in migraine patients, particular in those, who have failed several prophylactic therapies.

## Background

Migraine is considered one of the most prevalent, disabling conditions affecting millions of people worldwide, especially women within the age of 15-49 years [1]. A national population-based survey in Germany, conducted between October 2019 and March 2020, revealed that 14.8 % of women and 6.0 % of men met all of the diagnostic criteria for migraine [2]. Migraine can be classified as episodic or chronic, based on monthly headache days (MHD) and monthly migraine days (MMD); patients with fewer than 15 MHD have episodic migraine (EM) [3], and those with at least 15 MHD (and at least 8 MMD) for more than 3 months have chronic migraine (CM) [4]. Current treatment options for migraine include a combination of both acute treatment of headache attacks and prophylactic therapies. The latter is suitable for patients with more than four migraine attacks that cause disabilities for two or more days a month and cannot be controlled by the use of acute medication [5]. The majority of available prophylactic medications were originally developed for other conditions, such as hypertension, epilepsy, and depression. In epidemiological studies it was shown that the treatment with these often resulted in discontinuation already during the first 60 days due to a lack of efficacy or poor tolerability [6].

Calcitonin gene-related peptide (CGRP) has been shown to play a pivotal role in migraine pathophysiology [7]. A fundamental advance in migraine management has been the development of monoclonal antibodies targeting the CGRP pathway, representing the first selective prophylactic treatments based on the pathophysiology of migraine [8]. Erenumab, the first-in-class fully human monoclonal antibody targeting the CGRP receptor, showed efficacy and safety in large randomized clinical trials for preventive treatment of migraine patients with and without prior treatment failures [9–12]. Recently published real-world data have further confirmed the efficacy and tolerability profile of erenumab in patients suffering from migraine who failed prior prophylactic treatments [13–31]. However, these real-world publications were mainly single-centre investigations, comprised of small patient population or did not focus on the German population. Due to the requirements of the German Federal Joint Committee (Gemeinsamer Bundesausschuss = GBA) for reimbursement, mainly severely affected patients are treated so far, the prerequisites being the failure of at least five approved prophylactics or the contraindications to those. In CM patients, prior failure to onabotulinumtoxin A is additionally required [32]. This unique situation in Germany provides a particularly, severely affected patient population in clinical practice, which is not sufficiently investigated so far. Furthermore, direct experience from the treating physicians on patient profiles and treatment patterns of erenumab in daily routines have not been reported so far.

In TELESCOPE, the first real-world data was collected in a multi-centre setting across Germany in order to characterize the use of erenumab in clinical practice from the treating physicians’ perspective. An online survey was conducted to document physicians’ experiences regarding current patient profiles for CGRP antibody recipients, therapy approach and treatment responses with erenumab. A retrospective chart review was conducted to analyse data of migraine patients being treated with erenumab for at least three months, focusing on treatment effects and various aspects of patients’ quality of life (QoL).

## Methods

TELESCOPE was a German online survey, capturing the professional assessment of erenumab treatment from 45 headache specialists. The survey was accomplished between July 2019 and December 2019. The project consisted of two parts. In the first part, treating physicians reported experiences on treating patients with erenumab. In the second part, physicians consecutively documented at least 10 but not more than 30 of their patients that had received erenumab for ≥ 3 month via a retrospective chart review. A steering committee comprising of medical and scientific experts in neurology and migraine was established to provide independent advice and input into the development of the survey questionnaire. The participating physicians completed the 24 items of the standardized questionnaire and were asked to respond, supported by a comprehensive research of their patient files.

For the first part of the questionnaire, participating physicians shared their experience with erenumab (including their current therapy approach and treatment responses. The first four questions reflected the characteristics of the participating centres (e.g. number of headache and migraine patients treated). The following four questions evaluated physicians’ experiences in treating migraine patients; i.e. the current patient profile for the initiation of monoclonal antibody treatment, treatment management and general outcome of the treatment with erenumab. The latter included onset of treatment effect, reduction of MMD and headache intensity, improvement in QoL and improvement of typical migraine symptoms (e.g. sensitivity for light and noise, nausea/emesis, allodynia, and aura). Another question concerned physicians’ opinion on the most important criteria for therapy outcome evaluation. The options available were reduction of MMD by at least 30 % or 50 % reduction of headache intensity, change in QoL and reduction of typical migraine symptoms.

The remaining 15 questions of the questionnaire reflected the retrospective chart review. Individual patients’ demographics and treatment characteristics were collected anonymised including gender, diagnosis of EM and CM, health insurance status, number and type of previous, prophylactic, medical and non-medical treatments and data on erenumab treatment (current doses and adjustments). The survey also inquired the physicians’ opinion on patients’ satisfaction and treatment success. For the latter, physicians were asked how many patients they consider as “responders” according to their professional judgement, which was based on the reduction of headache days and the overall impression of their patients in order to reflect current treatment routine in Germany. The last 8 questions concentrated on migraine-specific parameters (MMD, MHD and monthly acute medication days, attack intensity, frequency and accompanying aura), patients’ global impression on disease improvement and migraine-related symptoms. The occurrence of accompanying aura symptoms was defined by the treating physicians.

To avoid a selection bias, physicians were instructed to collect retrospective data of a consecutive series of patients diagnosed with EM or CM. Data were handled confidentially and anonymity of patients was secured. Due to the research format, this study was exempted from ethics committee review. All data on physicians’ opinion and patient data were analysed descriptively. If needed for certain explorative analyses, the paired-samples t-test for continuous data were applied. *P*-values < 0.05 were considered statistically significant.

## Results

### Treating physicians’ perspectives

Headache specialists of 45 German headache centres participated in the online survey by completing a questionnaire and a comprehensive, retrospective chart review. At all sites, participating physicians had been treating 202 migraine patients in average during the previous three months; 12 sites (27 %) reported treating more than 300 migraine patients. In total, 9099 migraine patients have been treated by all participating physicians within the preceding three months, and of these, in average 19.1 % with monoclonal antibodies. Thereof, 78.6 % had erenumab for at least three months.

**Fig. 1:**
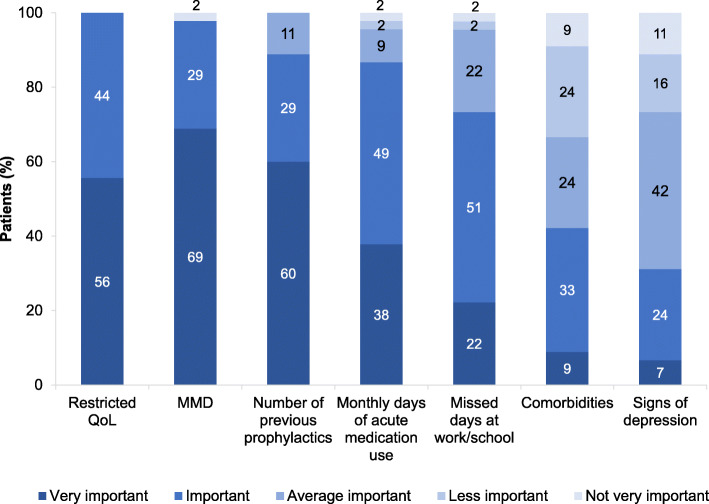
Reasons for initiation of treatment with a monoclonal antibody – physicians’ perspective. *N *= 45 (number of physicians); QoL=quality of life; MMD=monthly migraine days; Seven distinct reasons for the initiation of treatment with a monoclonal antibody were evaluated by the treating physician according to the level of importance

The current patient profile for treatment initiation was evaluated according to professional assessments of participating physicians. The four main reasons (rated as ‘very important’ and ‘important’) for treatment initiation with monoclonal antibodies were a restricted QoL (100 %; *n* = 45), number of MMD (98 %; *n* = 44), number of previously tried prophylactic therapies (89 %; *n* = 40) and monthly days of acute medication use (87 %; *n* = 39). Missed days at work/school were considered as important by half (51 %; *n* = 23) and comorbidities by a third (33 %; *n* = 15) of the physicians (Fig. 1).

**Fig. 2:**
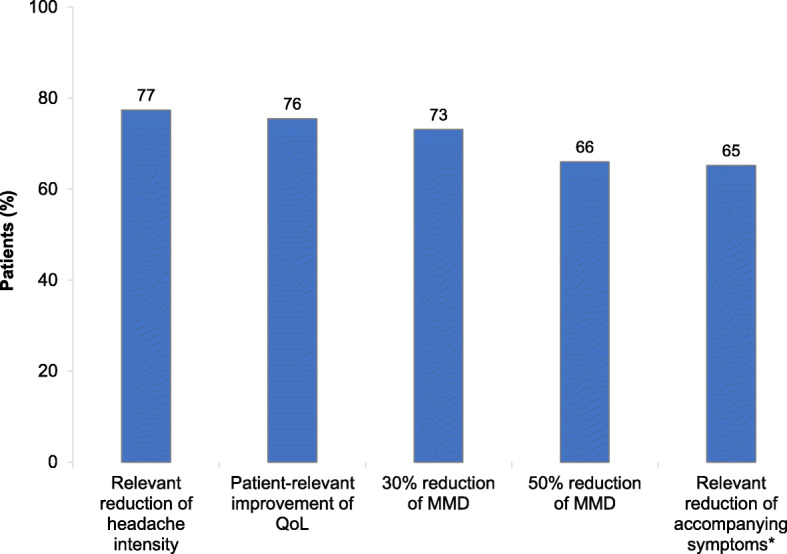
Characterization of the response to treatment in all erenumab responders since availability of erenumab from the physicians’ perspectives. *N* = 42 (number of physicians); QoL=quality of life (e.g., Headache Intensity Test, evaluated on a five-point Likert scale: HIT-6); MMD=monthly migraine days. *Accompanying factors include aura, sensitivity for light and noise, nausea and allodynia

Physicians’ file research of the total population, who have been treated for at least three months with erenumab revealed that the majority of patients (84 %) initiated treatment with 70 mg, whereas for 16 % the higher dose of 140 mg was chosen. They reported an onset of effect for 70 % ± 24 % of the population after the first injection, 20 % ± 15 % after the second and 8 % ± 10 % after the third antibody injection. In case of nonresponse by physicians’ discretion, 70 % of the physicians discontinued monoclonal antibodies after 3 consecutive months of treatment (data not shown). According to the experience of treating physicians, erenumab treatment had led to a relevant reduction in headache intensity in 77 % of the population and patient-relevant improvement of QoL in 76 %. During the data collection period in 2019, an at least 30 %-reduction in MMD was achieved in 73 % and a 50 %-reduction in 66 % of patients (Fig. 2). Nearly three quarters of the participating physicians (73 %) considered a reduction of MMD by 50 % and 30 % the most important criteria to evaluate treatment response in clinical practice. Further criteria were patient-relevant improvements of QoL (16 %) and reduction of headache intensity (4 %) (data not shown).

### Medical chart review of erenumab treated patients

In the second part of TELESCOPE, medical chart data of 542 consecutively selected erenumab patients were gathered anonymously. According to the retrospective analysis of the medical records, 85 % of the patients were female, 50 % were suffering from EM and 50 % from CM. About half of the patients (54 %) currently received an erenumab dose of 70 mg and 46 % the higher dose of 140 mg. Patients diagnosed with CM more frequently obtained the higher dose of 140 mg erenumab than patients with EM (58 % vs. 35 %). Throughout the previous treatment period, 48.2 % of all patients received a dose adjustment, which was more often applied to CM patients than to EM patients (57 % vs. 40 %). In 94 % of the patients who started with 70 mg (*N* = 252), the dose of erenumab was escalated from 70 mg to 140 mg. In some patients (8.3 %), who started treatment with 140 mg (*N* = 290), the dose was de-escalated to 70 mg (data not shown). At the time Telescope was conducted, patients have been treated with erenumab for 6.9 ± 2.7 months in average (Table 1).

**Table 1:** Patient characteristics

	Patients *N* = 542
Sex, n (%)	
Male	81 (15)
Female	461 (85)
Migraine type, n (%)	
Chronic migraine	271 (50)
Episodic migraine	271 (50)
Current dose of erenumab, n (%)	
70 mg	290 (54)
140 mg	252 (46)
Insurance status, n (%)	
Statutory health insurance	474 (87)
Private health insurance	68 (13)
Treatment duration with erenumab in month, mean (SD)	6.9 (2.7)

*SD* standard deviation

#### Previous migraine treatments

All patients (100 %) tried or were not suitable for in average 4.6 ± 1.4 prior medical prophylactic therapies, before initiating the treatment with erenumab. Previous treatments included mostly topiramate in 85.1 % of the patients, beta blocker in 80.6 %, amitriptyline in 72.0 % and flunarizine in 49.8 %. Onabotulinumtoxin A was additionally tried by 204 patients, all of whom were diagnosed with CM. Hence, 75.3 % of CM patients also failed previous treatment with onabotulinumtoxin A. The majority of all patients (74.9 %) also used non-medical therapies as prophylactic treatments with relaxation therapies being the most frequently used (Table 2).

**Table 2:** Previous prophylactic therapies of erenumab patients

**Medical therapies**^a^	Previous therapy N (%)	Contraindication against therapy N (%)
Topiramate	461 (85.1)	41 (7.6)
Beta blocker	437 (80.6)	68 (12.5)
Amitriptyline	390 (72.0)	81 (14.9)
Flunarizine	270 (49.8)	167 (30.8)
Onabotulinumtoxin A^b^	204 (37.6)	8 (1.5)
Valproate	107 (19.7)	249 (45.9)
Others	406 (74.9)	-
No previous therapy	9 (1.7)	
No contraindications	-	217 (40.0)
Number of previous therapies, mean (SD)	3.4 (1.4)	
Number of previous therapies and contraindications, mean (SD)	4.6 (1.4)	
**Non-medical therapies**		N (%)
Yes		406 (74.9)
No		136 (25.1)
Relaxation therapies		338 (62.4)
Physical exercise (sports)		270 (49.8)
Acupuncture		219 (40.4)
Psychological therapy of pain		168 (31.0)

*SD *standard deviation, *N *542 (number of patients)

^a^Multiple responses were possible

^b^Only patients with chronic migraine additionally received onabotulinum A (i.e. 75.3 % of CM patients)

#### Effects of erenumab treatment

**Fig. 3:**
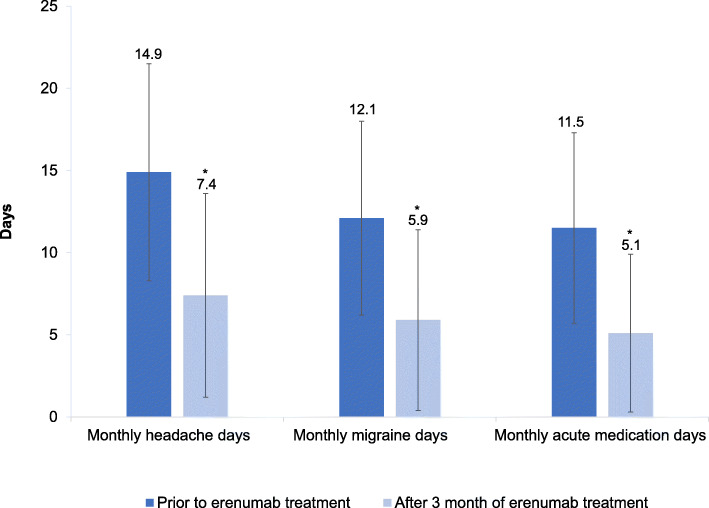
Headache-, migraine-, and medication days after 3 months of erenumab treatment – medical chart review (*N *= 542); MHD = monthly headache days; MMD = monthly migraine days; *P < 0.0001

After a three-month erenumab treatment period, MHD, MMD, as well as monthly acute medication days were significantly reduced in documented patients. Compared to baseline, the mean reduction in MHD after 3 months was -7.5 days (from 14.9 ± 6.6 to 7.4 ± 6.2; *P* < 0.0001). The mean reduction in MMD was -6.2 days after 3 month (from 12.1 ± 5.9 to 5.9 ± 5.5; *P* < 0.0001) and in monthly acute medication days -6.4 days (from 11.5 ± 5.8 to 5.1 ± 4.8, *P* < 0.0001) (Fig. 3). Of 542 migraine patients, 82.7 % were considered as “responders”, according to physicians’ professional judgement, which was based on the reduction of headache days and the overall impression of their patients. Treating physicians reported that 79.5 % of all patients were satisfied with erenumab treatment. Very high “responder” (88.6 %) and patient’s satisfaction (85.2 %) were evaluated in patients with EM (Table 3).

**Table 3:** “Responder” and patients’ satisfaction – physicians’ judgement

	Overall *N *= 542	EM *N *= 271	CM *N *= 271
“Responder”, n (%)			
Yes	448 (82.7)	240 (88.6)	208 (76.8)
No	94 (17.3)	31 (11.4)	63 (23.2)
Patient satisfaction, n (%)			
Yes	431 (79.5)	231 (85.2)	200 (73.8)
No	73 (13.5)	26 (9.6)	47 (17.3)
Uncertain	38 (7)	14 (5.2)	24 (8.9)

Treating physicians classified “responder” according to their professional judgement, which was based on the reduction of headache days and the overall impression of their patients

*EM *episodic migraine, *CM *chronic migraine

**Fig. 4:**
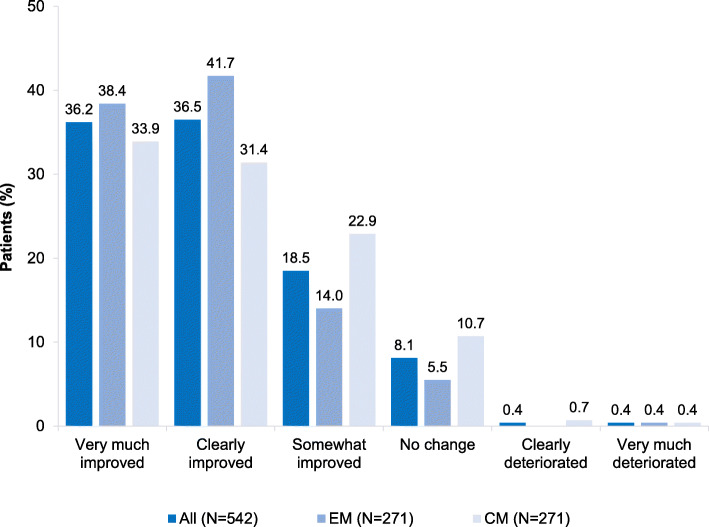
Global impression scale in migraine patients after 3 months of erenumab treatment – medical chart review (*N* = 542); EM = episodic migraine; CM = chronic migraine; Clinical global impression scale of improvement was defined by responses of very much improved, clearly improved, somewhat improved, no change, clearly deteriorated and very much deteriorated

**Fig. 5:**
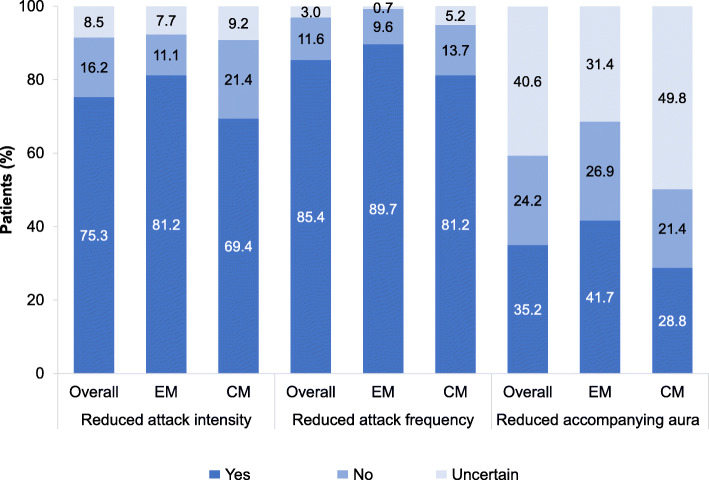
Effect of erenumab on migraine-specific headache parameters after 3 months of treatment – medical chart review (*N* = 542); EM=episodic migraine (*N* = 271); CM = chronic migraine (*N *= 271)

Based on the clinical global impression scale, treatment with erenumab for at least three months led to a general improvement (very much, clearly and somewhat improved) in 91 % of all patients. Patients with EM showed somewhat better improvements compared to those with CM; (very much (38.4 % vs. 33.9 %) and clearly improved (41.1 % vs. 31.4 %). Less than 0.5 % of all patients stated a deterioration of the disease during erenumab treatment (Fig. 4). Furthermore, relevant improvements in migraine-specific headache parameters and migraine-related symptoms were reported. In detail, 75.3 % of all patients had reduced headache intensity, 85.4 % reduced frequency of headaches and 35.2 % accompanying aura after three months of erenumab treatment. The presence of aura at baseline was not investigated. Improvements of attack intensity and frequency, were more pronounced in EM patients compared to patients with CM (81.2 % vs. 69.4 % and 89.7 % vs. 81.2 %). A reduction of accompanying aura was also an effect rather seen in EM patients than in CM patients (41.7 % vs. 28.8 %) (Fig. 5).

**Fig. 6:**
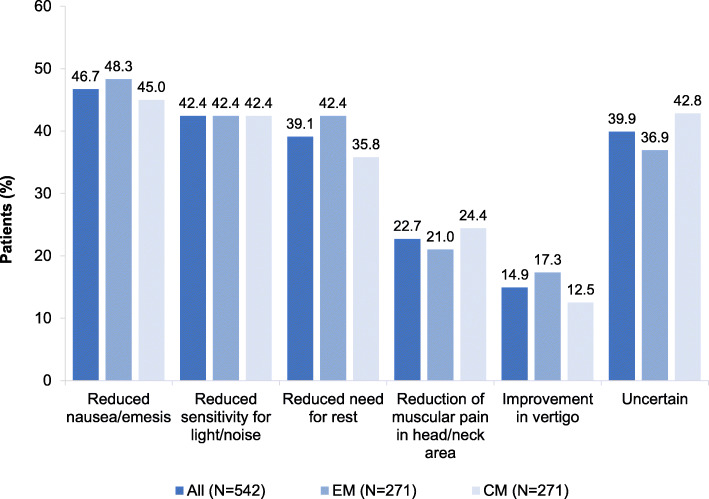
Effect of erenumab on migraine-related symptoms after 3 months of treatment – medical chart review. Multiple responses were possible; EM = episodic migraine; CM = chronic migraine

Regarding migraine-related symptoms, physicians reported that nausea and emesis were reduced in 46.7 % of all patients and sensitivity for light and noise in 42.4 % of patients three months after initial treatment with erenumab. About 39.9 % of patients were uncertain regarding improvement of these symptoms. The effects of erenumab on migraine-related symptoms were similar in both, EM and CM patients (Fig. 6).

## Discussion

TELESCOPE provides us with real-world evidence data on erenumab treatment gathered in a large German cohort. Three-month treatment with erenumab significantly decreased MMD, MHD and acute medication days, reduced headache frequency, intensity and frequency of accompanying aura, improved the patients’ clinical global impression scale and QoL in a patient population with several treatment failures. A reduction in nausea/emesis, light/noise sensitivity and the need to rest was reported in a remarkable number of patients. In addition, headache specialists reported the current patient profile and specific treatment patterns when prescribing erenumab. These findings captured the physicians’ professional perspective and judgement on the initial treatment outcome after three months of erenumab treatment in routine clinical practice and will thus be a valuable complement to the data obtained from randomized clinical trials. Furthermore, our data offer valuable information for experienced clinicians and might additionally provide guidance for healthcare specialists who have limited clinical experience with monoclonal antibodies in migraine management.

Treating physicians retrospectively documented data of 542 migraine patients. The vast majority of this cohort were members of a statutory (87 %) health insurance, while the remaining 13 % contributed to a private health insurance. This broadly reflects the current distribution of the German health care system with 10.5 % privately insured citizens [33]. Patients failed or had contraindications to an average of five prior, prophylactic migraine treatments before erenumab was prescribed. The most common discontinued prophylactic therapies were topiramate, beta blocker and amitriptyline. Three quarters of CM patients additional received onabotulinumtoxin A. This high number of treatment failures can be attributed to the regulations of the German GBA for the reimbursement of CGRP monoclonal antibodies by the statuary health systems and thus mirrors the current highly impacted patient population that receives erenumab treatment in Germany [32]. The data confirm that physicians follow the requirements of the statuary health system when prescribing monoclonal antibodies. Furthermore, as recommended by current guidelines, the majority of patients (75 %) also applied non-medical therapies for migraine prevention with relaxation techniques being used most frequently [34].

Three months treatment with erenumab led to a significant reduction of -6.2 MMD, -7.5 MHD, and -6.4 monthly acute medication days in this analysis. Data investigating erenumab treatment in large numbers of both CM and EM patients in real-world multi-centre settings are limited. However, our results are in the range of existing real-world evidence with erenumab gathered mainly in single-centre settings. Mean reductions in MMD of -3 to -13 days, MHD of -3 to -10 days and of -3 to -6 monthly acute medication days after three-month erenumab treatment in patients with an average of four up to eight prior prophylactic treatment failures were reported [13–20, 22–25]. All real-life data published so far complement and partly exceed the effects shown in clinical trials [8–10, 12].

Results of our analysis comprising of a substantial number of multiple treatment-refractory EM patients in particular support findings of the LIBERTY trial, in which a significant reduction of MMD and acute medication days was demonstrated. Under the treatment of erenumab, 30 % of all patients reached a ≥ 50 % reduction in MMD, compared to 14 % in the placebo group [12]. In our survey, participating headache specialists and neurologists considered 83 % of all patients as “responders” (89 % of EM and 77 % of CM patients). The estimation of the percentage of “responders was according to the physicians’ professional judgement, which was based on the reduction of headache days and the overall impression of the patients in order to reflect current treatment routine in Germany. Of note, only patients that actually had received 3 injections of erenumab over three months were analysed. Hence, patients who discontinued therapy early were not included in the “responder evaluation”. Even though current guidelines [35] recommend evaluation of the response to treatment with monoclonal antibodies only after three months, there may be reasons for early discontinuation, such as patients wish.

According to the chart review, 54 % of all patients currently received 70 mg of erenumab and 46 % the higher dose of 140 mg. CM patients more frequently obtained the higher dose of 140 mg than EM patients (58 % vs. 35 %). Physicians reported that in 84 % of the erenumab-treated population, treatment is usually started with the lower dose of 70 mg. This was reflected by the medical chart review as 94 % of patients who currently received 140 mg (*N* = 252) underwent a previous dose escalation from 70 mg to 140 mg. TELESCOPE was conducted approximately one year after marketing authorization in the European Union (July 2018) and market launch in Germany (November 2018). Thus, physicians might have been hesitant to start erenumab treatment with the higher dose. Available evidence suggests the preferred use of a 140 mg dose of erenumab over the 70 mg in EM and CM patients after several prior treatment failures [36, 37]. Our data and available evidence support that severely impacted patients with high numbers of prophylactic treatment failures and high numbers of MMD at baseline may warrant the higher dosage in order to obtain the full impact of erenumab. Further real-life studies could show whether the dose preference will change over time and experience with erenumab.

The physicians specified restricted QoL, high numbers of MMD and the number of previous, unsuccessful prophylactic treatments as the patient profile suitable for treatment initiation with monoclonal antibodies. This is in line with current German guidelines and reimbursement restrictions [32, 35]. Based on physicians’ clinical experience, erenumab treatment mediated a relevant reduction in headache intensity and patient-relevant improvement of QoL in more than three quarters of the treated population. The majority of the population (70 %) experienced treatment effects already after the first injection of erenumab. This early onset of erenumab action within the first weeks of treatment confirms data retrieved from controlled studies for CM and EM patients [38].

Besides the 50 % and 30 % reduction in MMD, physicians rated the importance of patient-relevant improvements of QoL, as one of the most important criteria to evaluate treatment responses in clinical practice. Erenumab-mediated improvements in QoL were reported in clinical practice, mainly using the Headache Impact Test (HIT) -6 score [13, 14, 16, 17, 19, 21, 22, 26].

The impact of migraine on QoL is often evaluated through the analysis of headache-specific validated questionnaires, in which other aspects of QoL are insufficiently covered. Complementary information, such as migraine-specific headache parameter, global impression, patients’ satisfaction and symptoms might better reflect the overall treatment benefit. According to the medical chart review, general improvements based on patients’ global assessment scale was seen in almost all patients (91 %). Furthermore, 85 % of EM patients and 74 % of CM patients were satisfied with erenumab treatment. This is in line with findings of another German real-life cohort in which the majority of patients (82 %) also reported to be satisfied with erenumab treatment [18]. Besides treatment efficacy, treatment satisfaction has been associated with drug adherence [39] and may be particularly important in therapy-refractory migraine patients as presented here. Another relevant therapeutic effect of erenumab treatment was evaluated by the reduction of attack intensity and frequency in over 80 % of EM and the majority of CM patients. In addition, patients reported reduction of accompanying aura (42 % of EM and 29 % of CM patients). These results are in line with the treating physicians’ experience reported within the first part of our survey, who stated that 77 % of their patients experience a relevant reduction of headache intensity and 65 % in accompanying symptoms, such as aura. In clinical practice, positive effects of treatment with erenumab on headache intensity have been shown mainly in CM patients [16, 18, 19]. With our data, we can support the existing real-world evidence in CM patients in a large cohort and extend it to EM patients.

To date, there are no well-proven treatments available to reduce accompanying aura. An aura prevalence of 35 % - 52 % [10–12] and 23 % - 35 % [15, 17, 30, 31] was reported in clinical trials and in real-world studies investigating erenumab, respectively. In our survey, physicians reported that 35 % of their patients had reduced accompanying aura, which was more pronounced in EM than in CM patients (41.7 % and 28.8 %). Interestingly, this is line with recent observations reported by Ament et al. 2021 for the treatment with another CGRP-monoclonal antibody [40]. Due to our survey design, baseline values of patients with aura for comparison are not available. Further data will be needed to understand the ratio between patients with aura at baseline and patients with a relief of these symptoms, and to understand the extent of the effect.

In the large global *My Migraine Voice* survey investigating disease burden of over 10,000 treatment-refractory migraine patients, symptoms beyond the headache phase were reported by the majority of patients. In detail, over three quarters of the investigated patients were suffering additionally from light sensitivity, sound sensitivity and nausea [41]. The authors suggested an unmet need of treatments for these symptoms. In our retrospective observation erenumab treatment addressed this need in a remarkable proportion of patients. In about half of the patients, nausea or emesis and in approximately 40 % sensitivity for light, noise and need for rest was reduced during erenumab treatment. Due to the retrospective character of the study an evaluation of the reduction was not feasible. But the question remains, how to accurately capture effects on non-pain symptoms in retrospective and prospective settings. Similar, albeit smaller, improvements in migraine symptoms with erenumab treatment were reported in another German real-life cohort [18]. Previous randomised trials did not focus on migraine symptoms; however, the ongoing EMBRACE study was designed to fully capture the effect of erenumab beyond MMD, on migraine-related symptoms. This study will provide further insights on the effect of erenumab treatment on non-pain symptoms and the quantification of the relief.

Some limitations need to be considered. Only specialized physicians participated in the survey implying a possible bias of the study population. The analysed population might differ from what other neurologists and general care specialists encounter in their daily routine. Patients at specialized centres are more likely to be refractory to therapies, as is reflected by the exhaustion of prior therapies and the high proportion of non-medical procedures. On the contrary, this can also be regarded as a strength, because especially in multiple refractory patients, physicians estimated the treatment as successful and well tolerated. Further limitations are the retrospective design and the type of reporting. Collected data are mainly based on patients’ medical charts and physicians’ opinion. Assessments of migraine-specific headache parameter, patients’ satisfaction, global impression and symptoms in real-world settings have been based on the patients’ perspective gathered by the treating physician. Thus, our findings may be affected by reporting and recall bias. The somewhat better treatment effects reported here, compared to randomized trials and other real-life studies might be explained by the high expectations regarding the effectiveness of the drug and the fast onset of erenumab, which makes a treatment response in patients apparent. But, similar observations were also reported for the efficacy of triptans in real-world setting, compared to the data from randomized trials [42]. Despite these limitations, TELESCOPE provides an approach that captures the effects of erenumab in migraine patients in clinical practice and supports treatment decisions.

## Conclusions

In TELESCOPE, we provide comprehensive retrospective real-world evidence in a large migraine cohort in Germany and in addition first real-life insights on physicians’ overall experiences with erenumab treatment in migraine patients. A restricted QoL, number of MMD and previous prophylactic treatments represent the main components of the current patient profile for CGRP monoclonal antibody recipients. Based on the assessment of physicians, erenumab reduced migraine symptoms in 65 % and increased quality of life in more than 75 % of their treated population. These reports were reflected by a medical chart review of 542 refractory migraine patients. The retrospective analysis revealed that three months of erenumab treatment significantly reduced migraine-specific headache parameter and increased QoL in this severely impacted patient population. According to the treating physicians’ professional judgement, the vast majority of their patients did benefit from and were satisfied with the treatment. The results of this survey show positive experiences of treating physicians with the use of erenumab in everyday clinical practice.

## Data Availability

The datasets used and analysed during the current study are available from the corresponding author on reasonable request.

## Abbreviations

CGRP: Calcitonin gene-related peptide receptor
CM: Chronic migraine
EM: Episodic migraine
HIT-6: Headache Impact Test
MMD: Monthly migraine days
MHD: Monthly headache days
SD: Standard deviation
QoL: Quality of life
